# Lupus Anticoagulant Detection under the Magnifying Glass

**DOI:** 10.3390/jcm12206654

**Published:** 2023-10-20

**Authors:** Angelo Claudio Molinari, Tiziano Martini, Laura Banov, Antonella Ierardi, Marzia Leotta, Alessandra Strangio, Rita Carlotta Santoro

**Affiliations:** 1 Thrombosis and Hemostasis Unit, IRCCS Istituto Giannina Gaslini, 16147 Genova, Italy; aclaudiomolinari@gaslini.org (A.C.M.); laurabanov@gaslini.org (L.B.); 2 Immuno-Haematology and Transfusion Medicine, Center for Congenital Bleeding Disorders, Cesena General Hospital, 47521 Cesena, Italy; 3 Hemostasis and Thrombosis Unit, Azienda Ospedaliero Universitaria Dulbecco, 88100 Catanzaro, Italy; antonellaierardi86@gmail.com (A.I.); marzialeotta@virgilio.it (M.L.); alestr87@gmail.com (A.S.); ritacarlottasantoro@gmail.com (R.C.S.)

**Keywords:** lupus anticoagulant, antiphospholipid antibody syndrome, thrombosis, anticoagulation, activated partial thromboplastin time

## Abstract

Diagnosis of antiphospholipid syndrome (APS) requires the presence of a clinical criterion (thrombosis and/or pregnancy morbidity), combined with persistently circulating antiphospholipid antibodies (aPL). Lupus anticoagulant (LA) is one of the three laboratory parameters (the others being antibodies to either cardiolipin or β2-glycoprotein I) that defines this rare but potentially devastating condition. For the search for aCL and aβ2-GP-I, traditionally measured with immunological solid-phase assays (ELISA), several different assays and detection techniques are currently available, thus making these tests relatively reliable and widespread. On the other hand, LA detection is based on functional coagulation procedures that are characterized by poor standardization, difficulties in interpreting the results, and interference by several drugs commonly used in the clinical settings in which LA search is appropriate. This article aims to review the current state of the art and the challenges that clinicians and laboratories incur in the detection of LA.

## 1. Introduction

The term “lupus anticoagulant” (LA) was coined by Feinstein and Rapaport in 1972, to designate an acquired inhibitor of blood coagulation found in the plasma of patients with systemic lupus erythematosus (SLE) [1]. LA indicates a heterogeneous family of autoantibodies directed against complexes formed by negatively charged phospholipids (PL) and proteins such as prothrombin, β2-glycoprotein-I (β2-GP-I), and others [2].

Given the absence of population-based studies, the true prevalence of antiphospholipid-antibody positivity (LA), anticardiolipin antibodies (aCL), or anti-β2 glycoprotein-I (β2GPI) antibodies in the general population is not known [3], but according to some authors the prevalence ranges between 1 and 5% [4].

Tests for aPL are positive in approximately 13% of patients with stroke, 11.5% with myocardial infarction, 9.5% of patients with deep vein thrombosis, and 6% of patients with pregnancy morbidity [4]. The absolute risk of a first thrombosis in antiphospholipid-antibody–positive patients who do not have other risk factors is reported as less than 1% per year. As arterial and venous thrombotic events in antiphospholipid-antibody-positive patients are often multicausal, the annual risk of a first thrombosis in patients with persistent antiphospholipid-antibody profiles and a systemic autoimmune disease or additional thrombotic risk factors may be as high as 5% [4].

LA is one of the three laboratory criteria (Sydney criteria) defining antiphospholipid syndrome (APS) [5], a systemic autoimmune disease defined by thrombotic or obstetrical events that occur in patients with persistent antiphospholipid antibodies [6]. The other two criteria defining APS are the presence of anti-cardiolipin antibodies (aCL) and anti-β2-glycoprotein-I antibodies (aβ2-GP-I). For the search for aCL and aβ2-GP-I, traditionally performed by solid-phase immunoassays (ELISA), several different assays and detection techniques are currently available, thus making these tests relatively reliable and widespread [7]. In contrast, LA detection is based on functional coagulation assays that are characterized by poor standardization, difficulty in interpreting the results, and interference by several drugs commonly used in the clinical setting in which LA search is appropriate [8]. The drugs that have most commonly been related to false-positive LA detection are summarized in Table 1. A recent guidance from the Scientific and Standardization Committee for lupus anticoagulant/antiphospholipid antibodies of the International Society on Thrombosis and Haemostasis (ISTH) [9] focused on methodological aspects of LA search and on interpretation of the results; nevertheless, the LA diagnosis maintains some pitfalls that should be examined to increase clinicians’ awareness.

## 2. Indications to the Tests: Patients’ Selection and Timing of Testing

Testing for LA is appropriate only in those patients in which there is a reasonable suspicion of APS [9]: for the intrinsic weaknesses of these functional tests, an accurate selection of patients is essential to avoid incidental findings, which are potentially able to disorient the clinician; regardless, it has been demonstrated that asymptomatic persistent carriers of LA and of the other antiphospholipid antibodies have a higher risk of future thromboembolic events [11,12]. The timing of testing is a crucial point to avoid situations that are potentially prone to misinterpretation of the results:

⮚ Current guidelines recommend avoiding LA testing during an acute thrombotic event or an acute episode (e.g., an infection) [9], because of the interference of raised levels of coagulation factors (factor VIII) and of C-reactive protein on LA assays (see Section 3.2, “Interference”).
⮚ During pregnancy, many coagulation factors are physiologically increased (especially factor VIII) [13], making LA testing results’ interpretation difficult; ISTH recommends that in this setting the results should be taken into consideration with caution [9] (see Section 3.2, “Interference”).
⮚ Ideally, LA testing should be performed in patients not taking any anticoagulant drug [9]; the search for LA in anticoagulated patients is currently a matter of great debate, because in these patients, anticoagulant therapy is often started very soon and the possibility of performing LA testing while on anticoagulation drugs assumes importance. A recent guidance of the ISTH Scientific and Standardization Committee for lupus anticoagulant/antiphospholipid antibodies faced this argument [14], concluding that LA detection during anticoagulation remains a challenge. The LA search in anticoagulated patients is adequately discussed in Section 5 (“LA detection in anticoagulated patients”).

Table 2 indicates the settings in which LA search should be performed or could be considered, and the most appropriate timing for this search [9].

## 3. Preanalytical Phase

### 3.1. Sample Characteristics

The International Society on Thrombosis and Haemostasis (ISTH) and the Clinical and Laboratory Standard Institute (CLSI) produced guidelines that provide detailed information on the preanalytical phase of LA testing [9,15]. Venous blood must be collected into 0.109 mmol/L sodium citrate with blood/anticoagulant ratio 9/1 and rendered platelet-poor (final platelet count < 10 × 10^9^/L) by double centrifugation (2000× *g* for 15 min or similar) at room temperature. Filtration by cellulose acetate filters is not recommended, although it is very high performing in removing all the platelets, because it causes the loss of von Willebrand factor and other coagulation factors [16]. A correct centrifugation (apparently a banal procedure) is essential for the success of the tests, because the activated platelet surface expresses negative-charged PL, which is able to bind the antiphospholipid antibodies, providing a false-negative result, especially with low-titres antibodies [17]. This problem can be exacerbated using frozen plasma (in situations in which an immediate analysis of the sample is not possible) because repeated freeze/thaw cycles may disrupt the platelet membrane, releasing an excess of PL [18]. For this reason, the aforementioned guidelines suggested only one cycle of freezing and thawing, freezing the plasma within 4 h of the collection, rapidly thawing it at 37 °C for 5 min in water bath by total immersion, and analyzing it within 4 h; according to CLSI guidelines [15] the frozen plasma can be stored for 14 days at −20 °C and is stable for 6 months at −70 °C. A recent study, performed with respect to the CLSI guidelines, reported the persistence of dilute Russell’s viper venom time (dRVVT) and silica clotting time (SCT) positivity before and after one freezing/thawing cycle (thus demonstrating the stability of frozen plasma) [18].

### 3.2. Interference

During an acute thrombotic event, the raising of factor VIII levels can shorten activated partial thromboplastin time (aPTT), producing false-negative LA screening by aPTT tests [19], while dRVVT is not influenced by factor VIII levels, as factor X is directly activated by the diluted Russell’s viper venom [7]. Increased levels of factor VIII can also be found in pregnancy, cancer, surgery, and acute episodes such as inflammation and infection. In these situations, the results of LA testing should be taken into consideration with caution. The association of LA positivity with a phlogistic condition, not always accompanied by a clinical APS phenotype, was recently confirmed in patients with COVID-19 [20,21,22]. C-reactive protein (an acute-phase reactant), through its affinity with the PL present in the reagents, can prolong PL-dependent clotting tests and lead to false-positive LA tests [23]. Furthermore, several drugs (antibiotics, antiarrhythmics, chlorpromazine) and vaccines (hepatitis B) can occasionally be associated with a LA positivity [24]. Anticoagulants of any species are able to prolong the clotting times of PL-dependent tests, leading to difficulty in interpreting LA testing results. LA search while on anticoagulation drugs is discussed later (Section 5, “LA detection in anticoagulated patients”).

## 4. Lupus Anticoagulant Detection Procedure

### 4.1. General Principles: Three Steps Approach

LA detection is based on the in vitro functional behavior of these autoantibodies, which are able to affect some coagulation assays, producing a prolongation of clotting times. The heterogeneity of these antibodies and the variability of the effect they provoke in several coagulation assays explain why there is not a single test that is sensitive to all the LA, but it is necessary to use a combination of several assays for a correct diagnosis [25]. The so-called “three-step approach” consists of performing a sequence of three coagulation assays:

1. A screening assay, namely a PL-dependent test: aPTT, dRVVT (as recommended by ISTH guidelines [9], see above); if present in the patient plasma, LA is able to bind and inhibit PL, prolonging the clotting time beyond the upper limit of the reference range;
2. A mixing assay, in which the coagulation test is repeated on a mixture of normal plasma and the patient’s plasma; if LA is present in the patient plasma, the increase of coagulation factors provided from the normal plasma will not be able to correct the prolongation of the clotting time;
3. A confirmatory assay, in which the coagulation test is repeated while increasing the PL concentration; if LA is present in the patient’s plasma, the excess of PL is able to quench these antibodies, causing a shortening of the clotting time.

Table 3 shows the details of diagnostic tests of the three-step procedure for LA.

This sequential approach allows one to exclude the situations in which coagulation abnormalities other than LA are present, narrowing down the diagnostic possibilities until the presence of LA is confirmed or excluded. Therefore, this stepwise procedure can reduce costs, avoiding unnecessary mixing and confirmatory tests if the screening test is normal; however, in daily practice, screening and confirmatory tests are often performed at the same time, followed (if necessary) by the confirmatory test. The (possible) weak points of the three-step method and its possible modifications are discussed above (Section 8, “Diagnostic Algorithms”).

### 4.2. Choice of Assays

The most recent ISTH guidelines [9] recommend using at least two tests as screening, based on different principles:

1. A LA-sensitive test derived from aPTT (such as SCT, silica clotting time);
2. The dRVVT (dilute Russell viper venom time).

The aPTT is based on activation of the intrinsic pathway. Its sensitivity to LA depends on the combination of two aspects: the type of activator and the concentration of PL. It is recommended to select an aPTT that uses silica as activator [9]. Silica clotting time (SCT) is a phospholipid-dependent test that contains colloidal silica as activator and a very low concentration of PL [37], thus making it very sensitive to LA. Ellagic acid has shown acceptable sensitivity in some aPTT reagents [26]. Further information about aPTT is available in [38].

Dilute Russell viper venom time utilizes a potent activator of factor X, the Russell’s viper venom. Russell’s viper, also known as Daboia russelii, is a venomous snake species, a member of the Viperidae family, found in South Asia, including India, Pakistan, Sri Lanka, Bangladesh, and other neighboring countries. Its potent venom interferes with the blood clotting process as it contains enzymes that directly activate some coagulation proteins (factor X, factor V, prothrombin, fibrinogen, and plasminogen [39]), making this test sensitive to deficiencies (congenital or acquired) of these factors, while it is independent of a deficiency of intrinsic pathway factors (XII, XI, IX, VIII) [37]. However, up to 20% of patients with factor VIII inhibitors can show a positive dRVVT test [40]. dRVVT is recommended for its specificity and robustness [9].

Both aPTT-based assay and dRVVT should be performed in highly standardized conditions, and their results should be expressed as the ratio between a patient’s clotting time and a normal pooled plasma (NPP) clotting time [8]. As aPTT and dRVVT are both positive in only a small fraction of patients, the recommendations indicate considering the LA screening positive whenever one of the two tests is positive.

Beyond aPTT and dRVVT, several phospholipid-dependent assays exist but are not recommended because of their limited commercial availability, poor reproducibility, and variability in reagents’ composition (Table 3).

Moreover, there is one relatively novel test that needs to be mentioned in the LA research that is based on the analysis of the clot formation curve: clot waveform analysis (CWA). CWA is performed using a coagulation analyzer that measures the changes in light transmission as a blood clot forms. The clot formation curve is then analyzed for specific features that are characteristic of LA. CWA has been shown to be a sensitive and specific test for LA detection. It is also a relatively simple and inexpensive test to perform, making it a potential alternative to traditional LA testing methods, such as the APTT and the dRVVT. CWA can be used to detect LA in patients with a prolonged aPTT or dRVVT and has the potential to revolutionize the diagnosis of LA. However, it is little used in laboratories and its clinical utility is still being evaluated [41]. Therefore, it is not included in this review.

### 4.3. Screening Tests

The aim of the screening test is to evidence a prolongation of clotting time that could be related to the presence of LA. Because of the in vitro competition between LA and coagulation factors for phospholipid-binding sites, the phospholipidic component of the test is importantly diluted to accentuate the inhibitory effect of any LA antibody present [25]. The characteristics of the aPTT and the dRVVT are crucial for LA detection. They exist under many different commercial brands, which differ from each other in their composition of phospholipids and activators; these differing compositions determine differences in the sensibility and specificity of the procedure: a general principle is that reagents containing a low concentration of phospholipids are more sensitive to LA [8]. In addition, the representativeness of the different phospholipids classes can influence the assay performance [42,43,44]: the higher the relative content of phosphatydilserine, the less the sensitivity of the assay to LA. The specificity of LA screening test is hard to define, because of the difficulty of finding a test able to certainly individuate the “real” true positives (the “true LA”). There is some evidence that the association between LA detection and clinical events is stronger for dRVVT than aPTT [45]. Currently, there are no strict recommendations about the best composition of the phospholipids of the screening assay. The role and the importance of the activators have been already discussed above (Section 4.2).

The CLSI guideline states that the dRVVT is the preferred test for LA detection, due to its high sensitivity and specificity. The aPTT and SCT are less sensitive than the dRVVT for LA detection [15].

### 4.4. Mixing Tests

A mixing test with screening reagent must be performed if the screening test clotting time is prolonged. A mix should be obtained utilizing a 1:1 proportion of patient plasma and pooled normal plasma (PNP), without incubation, within 30 min [9]. In theory, the coagulation factors contained in PNP will correct the prolongation of the screening test, restoring a clotting time that falls in the range of normality, if affected by a deficiency of one (or more) factors; if an inhibitor is present, the clotting time will remain prolonged [46,47]. PNP is very important for the standardization of the mixing test; it should be platelet-free, its content of each coagulation factor should be close to 100%, and it should be obtained from at least 20–40 healthy donors (males and females) [9,48]. PNP can be prepared “in house”, by mixing normal plasmas in a plastic receptacle, splitting the mix into small aliquots, and putting them into plastic tubes. The aliquots will then be rapidly frozen for storing at −70 °C (a temperature that ensures the stability of each coagulation factor for about 6 months). The aliquots of PNP can be thawed just before performing the test by rapidly exposing them at 37 °C in a thermostatic bath, which is gently tilted, and used within 2 h. There are several commercial lyophilized plasmas that show the same characteristics of home-made PNP and can be employed for performing the mixing test [49].

### 4.5. Confirmatory Tests

The confirmatory test consists of the execution of a screening test that has been made insensitive to LA: this is obtained by employing concentrated phospholipids in the reagent, to provide an antigen excess that is able to quench LA, abolishing the competition between LA and coagulation factors for phospholipids binding sites. This will result in a shortened clotting time of the screening test, which will fall into the reference range [25]. If an inhibitor other than LA is present, it will not be affected by the elevated phospholipids concentration, resulting in a confirmatory test that is unvaried compared to the screening test.

An alternative to adding an excess of phospholipids can be the use of reagents whose phospholipids component is innately LA-insensitive [50,51]: hexagonal phase phosphatidylethanolamine, purified inosithin, or phosphatydilserine [43,44,52,53].

ISTH guidelines recommend that confirmatory test(s) must be performed by increasing the concentration of phospholipids used in the screening test(s) [9].

### 4.6. Interpretation of Results

Clotting times of screening, mixing, and confirmatory procedures are prone to inter- (between same-principle tests offered by different manufacturers) and intra-assay (between batches of the same reagent) variability [25]; for this reason, it is recommended to normalize a screening, mixing, and confirmatory clotting time, generating a ratio between test clotting time and NPP clotting time [9,36,48,54,55].

#### 4.6.1. Screening Tests

Results of screening tests are suggestive for the presence of LA (or coagulation factor deficiency) if the clotting time ratio for the patient is higher than the cut-off.

#### 4.6.2. Mixing Tests

The most widely used way to interpret mixing time results is to use the index of circulant anticoagulation (ICA, also called Rosenberg–Rundle index, often shortened to Roxner index), which can be calculated based on the clotting time of patient plasma, NPP, and mixture [8]:

ICA = [(CT _mix_ − CT _PNP_)/CT _patient_] × 100

If ICA is above the cut-off, the mixing test is suggestive of a circulating anticoagulant (LA or a factor-specific inhibitor). Nevertheless, ICA, originally developed for LA detection and later exported to the interpretation of other mixing tests in hemostasis [56], has recently been demonstrated to be less sensitive to LA than a mixing-test-specific cut-off expressed as a normalized ratio [57,58,59]. Therefore, ISTH guidelines recommend a mixing-test-specific cut-off over ICA [9].

#### 4.6.3. Confirmatory Tests

Phospholipids’ dependence on clotting time prolongation is evidenced by calculating the percent correction of the screening by the confirmatory procedure:

%Correction = [(CT _screening_ − CT _confirm_)/CT _screening_] × 100

If percent correction is higher than the cut-off, the result is indicative of the presence of LA [8,25].

#### 4.6.4. Integrated Assays

Integrated assays perform screening and confirmatory tests in the same procedure. Results are expressed as a ratio between screening and confirmatory (higher than cut-off for LA, lower than cut-off for factor deficiencies). Many laboratories are now adopting these integrated assays, omitting the mixing. The uncontested advantage of this approach is the standardization of the tests, greatly reducing operator intervention. Nevertheless, in some cases LA behaviour is peculiar, and it cannot be individuated if mixing is not performed [8]. The “lupus cofactor” phenomenon identifies a setting in which LA needs a plasmatic cofactor (probably prothrombin) to express its inhibitory activity; if the cofactor is not provided by NPP, LA cannot be diagnosed [60]. Currently the incidence of this phenomenon among LA-positive patients is not exactly known, but it is thought to be rare.

#### 4.6.5. Cut-Offs

Results of LA testing must be compared with adequate cut-off values for each step of the procedure (screening, mixing, and confirmatory).

ISTH guidelines expressly recommend not using cut-off values established elsewhere, but using “in house” cut-off values deriving from a sufficiently large population of healthy individuals (at least 120): the value of cut-off is determined to be the 99th percentile [9]. CLSI and BSH guidelines [15,36], on the other hand, employ a “parametric” approach assuming a Gaussian distribution of LA results, using a reference range of ±2 standard deviations from the mean value (even if some works have shown that these values do not respect the Gaussian probability model [61,62]). The first approach is characterized by an increased specificity (that reduces false positives) and a statistically inevitable reduction of sensitivity (partially counterbalanced by the use of two assays) [7]. The second approach moves toward a balance between sensitivity and specificity [25].

In clinical practice, the enrolment of 120 subjects is difficult (especially for small laboratories); an alternative could be the use of manufacturer-provided cut-offs (assuming that these derive from a correct procedure in terms of population selection, statistical method, etc.). Their accuracy must be checked in any case by testing a smaller population of healthy subjects (20 or 40) [8]. ISTH guidelines affirm that this approach is possible “if manufacturers provide cut off values established in accordance with guidelines and by appropriate statistical models using a sufficiently large donor population” [9]. CLSI also allows this procedure [15], only if an adequate comparison of the suggested cut-off values is performed with a healthy (limited) population. These guidelines also consider the possibility of acquiring cut-offs established in other laboratories, even if evidence shows that there is a great inter-laboratory variability even when using the same analytic platform [61,63].

## 5. LA Detection in Anticoagulated Patients

Anticoagulants are able to prolong the clotting time of screening, mixing, and confirmatory tests, thus making the interpretation of these results difficult for the clinician (Table 4).

Until a few years ago, LA detection during the treatment of an acute thrombotic episode was not an essential matter, as it was possible to delay it until the discontinuation of a regular course of VKA (vitamin K antagonists) therapy. The therapeutic option represented by DOACs (direct oral anticoagulants), currently the standard of care of anticoagulation for the vast majority of patients, brought to light the importance of LA detection before starting anticoagulant treatment. Recent clinical trials of patients with APS randomized to receive VKA or rivaroxaban (a direct factor Xa inhibitor) showed a significant excess of thrombosis recurrence in rivaroxaban patients [65,66]. The premature interruption of the TRAPS trial [65] led the EMA (European Medicines Agency) to warn against the use of DOACs in APS patients, recommending the exclusion of APS in patients with acute thrombosis after unspecified causes for whom treatment with a DOAC is indicated, shifting anticoagulation to VKA if there is an anti-phospholipids-antibodies positivity [67].

A recent guidance from the Scientific and Standardization Committee (SSC) for lupus anticoagulant/antiphospholipid antibodies of the ISTH [14] stated that “LA testing in patients on anticoagulation should be undertaken with the cognizance that anticoagulants may prolong the clotting time of the tests used for LA detection and that this effect may give rise to false-positive or false-negative LA”.

The effects of several anticoagulant drugs on LA detection and the possible strategies for diagnosing LA in anticoagulated patients are discussed in the following paragraphs.

### 5.1. Unfractionated Heparin (UFH) and Low-Molecular-Weight Heparin (LMWH)

Patients on heparin do not need an interruption of treatment, if the assays employed for LA detection contain chemical agents able to neutralize the effect of UFH and LMWH, such as heparinase or polybrene (manufacturers should also specify the maximum concentration of heparin that is quenched, usually 0.8–1.0 IU/mL). False positives have been described for aPTT and dRVVT even when utilizing neutralizers [68,69,70]. However, ISTH SSC guidelines recommend measuring anti-factor-Xa activity in heparin-treated patients, to verify if the UFH or LMWH effect is at therapeutic interval; in this case, LA testing can be performed using assays containing heparin neutralizers [14].

On the other hand, performing a thrombin time test, which is very sensitive to UFH (less to LMWH) but not to LA, also helps to rule out the presence of UFH in the patient sample [8].

A practical expedient could be also taking the blood sample at least 12 h after the administration of heparin.

### 5.2. Vitamin K Antagonists (VKA)

All the assays for LA detection, except those employing snake-venom-derived prothrombin activators, are affected by VKA [25]. These drugs cause an acquired deficiency of factors II, VII, IX, and X that can give false positives in screening tests and false negatives in mixing tests [7]. Mixing patient plasma with NPP can correct these factor deficiencies, especially if patient INR (international normalized ratio) is <3.0 [8]: for this reason, CLSI, BSH, and past (2009) ISTH guidelines [15,33,36] consider the possibility of 1:1 mixing with NPP before testing. This approach can lead to potential false negatives, as LA activity also diluted. Moreover, the degree of correction depends on the reagents employed [14,71,72]. For this reason, current ISTH guidelines [9] consider the mixing procedure to be unreliable for VKA-treated patients.

Possible strategies to address this interference are: a temporary switch to LMWH, prior to LA testing, and subsequently changing back to VKA (a procedure requiring a lot of time, many INR checks, and potentially posing a high bleeding risk [14]); and the use of the combination of TSVT/ET (Taipan snake venom time/ecarin time), which in an international multicenter multiplatform study demonstrated good sensitivity and specificity in LA detection in VKA-treated patients [28], but is not routinely utilized in laboratories at present (see Table 4).

### 5.3. Direct Oral Anticoagulants (DOACs)

Direct factor Xa inhibitors (apixaban, rivaroxaban, and edoxaban) and the direct factor IIa inhibitor dabigatran are able to induce effects on coagulation that are present also at drugs’ trough levels (and are not sensitive to heparin neutralizers). All the assays for LA detection, except those employing snake-venom-derived prothrombin activators, are affected by DOACs [25]. These drugs can give both false-positive and false-negative results; all DOACs prolong dRVVT in screening and confirmatory procedures, with rivaroxaban affecting screening more than confirmatory (increasing potential false positives) and opposite apixaban action (increasing potential false negatives) [73,74,75,76,77,78].

One possible strategy is the use of absorbents, active charcoal compounds able to remove DOACs molecules from plasma and therefore eliminate interference in PT (prothrombin time), aPTT, dRVVT, and SCT [79]. Some products of this category are currently available as tablets (DOAC-stop^®^, DOAC-remove^®^) [80,81,82,83]. After mixing absorbents and patient plasma, a short incubation and a centrifugation of the mixture are performed, and the supernatant plasma (theoretically deprived of anticoagulant) is used for LA testing [8]. Alternatively, filtration techniques are available to remove DOACs from the plasma sample [84,85,86]. Both removal strategies can provide an incomplete drug removal or influence the clotting times, resulting in possible false positives and false negatives in LA testing [81,82,85,86]. DOAC removers must be used only in patients known to be undergoing therapy with these drugs [14]. Furthermore, DOAC levels after removal should be measured to ensure a (theoretic) reliability of LA testing [25].

An alternative method to resolve these interferences is the aforementioned combination of TSVT (Taipan snake venom time) and ET (ecarin time), which can also be useful for patients treated with direct factor Xa inhibitors but not for those in therapy with dabigatran [28].

A practical approach to face the problem of LA testing in a young patient with first unprovoked (or provoked by a mild risk factor) venous thromboembolism already on anticoagulant treatment was given by Pengo [87]:

1. Ask the patient for personal and familiar history of autoimmune diseases;
2. Search for anti-cardiolipin and aβ2-GP-I antibodies (not influenced by anticoagulants):
  ⮚ If negative: DOACs can be utilized;
  ⮚ If positive: go to step 3;
3. Test for anti-phosphatydilserine/prothrombin antibodies (aPS/PT), which can be considered a surrogate of LA as these antibodies generally present in triple-positive patients (determining the so-called “tetra-positivity”) and are responsible for the majority of LA activity [88,89].

Table 5 lists the main recommendations in guidelines regarding LA testing in anticoagulated patient.

## 6. LA and Other Coagulation Factors Assays

Other coagulation assays that use aPTT-based methods, as one stage measurement (OSA) [90] for factors VIII, IX, XI, and XII, may show interference due to LA presence, resulting in falsely low factor activity [91,92,93,94,95,96,97,98]. In these cases, some clotting factor activity is usually measurable, determining a non-parallelism between patient plasma and standard plasma during OSA performance [97]. Nevertheless, a very low or undetectable factor activity, without non-parallelism, has been reported in several cases [95,97,98,99]: these effects can be seen in all OSA factor assays and pose the problem of differential diagnosis with both a multiple factor deficiency and a factor-specific inhibitor. A fundamental aid in these circumstances comes from performing chromogenic factor assays (CSA) [90], which do not suffer from these interferences, showing normal clotting factor activities [92,96,97].

## 7. LA and Other Inhibitors

LA can be hard to distinguish from other antibody-mediated coagulation alterations, such as factor VIII autoantibodies characterizing acquired haemophilia A (AHA). Even if these inhibitors require incubation to express their activity (2 h at 37 °C), if they are present at very high titre, they immediately neutralize factor VIII of NPP in the aPTT mixing procedure, thus making them indistinguishable from LA [8]. Furthermore, some factor VIII inhibitors show a behavior similar to LA in confirmatory tests [100]. Importantly, LA can cause false positives in Bethesda assays [40]. dRVVT is very useful for excluding the presence of LA, as this test is less sensitive to factor VIII deficiency; however, it has been demonstrated that up to 20% of patients with factor VIII inhibitors show a positive dRVVT [40].

Italian, British, and international guidelines on the diagnosis of AHA indicate the need to exclude the presence of LA and investigate an isolated prolonged aPTT that does not correct with a mixing test [101,102,103].

## 8. Diagnostic Algorithm(s)

The “traditional” diagnostic algorithm, structured by the sequence of screening, mixing, and confirmatory procedures, recently became a matter of debate because of the pitfalls of mixing tests, which are challenging the central role of this assay in LA diagnostic work-up. The principal limitation regarding mixing is the unavoidable dilution effect on the antibody titer, which in some cases can make the LA undetectable, leading to false-negative results [104]. False-negatives mixing tests can also arise for reasons other than dilution, such as reagents’ sensitivity and specificity to LA: some LAs, undoubtedly positive in screening tests with both aPTT and dRVVT, show positivity in the mixing for one test but not for the other [105,106]. Finally, NPP characteristics can influence the results of mixing tests, as some NPPs have shorter clotting times, requiring a stronger LA for a mixing test be positive [107]. Moving from these premises, British Society of Hematology (BSH) guidelines [36] state that mixing tests improve specificity and, in the absence of other causes of prolongation of clotting times, samples that give positive results in screening and confirmatory tests on undiluted plasma should be considered positive. CLSI guidelines [15] move forward, giving a new priority order to the three steps: screening, confirmatory, and then mixing only if it will enhance diagnostic decision making. ISTH and BSH guidelines recommend that mixing studies should be considered an essential component of the LA diagnostic pathway, to identify the inhibitory nature of LA [9,36]. Besides the “classical” algorithm, an “iterative” algorithm can be found, in which an elevated screening test is followed by the confirmatory test. If it is normal and the % correction is above the cut-off, LA detection is positive; if the confirmatory test is prolonged and the % correction is above the cut-off, a mixing test is performed; if mixing is prolonged, LA detection is positive [25]. Integrated tests, as already said, concomitantly perform screening and confirmatory procedures, showing LA positivity if the ratio between these assays is elevated.

## 9. Reporting of Results

Given that LA test interpretation is so rich in complexity, even for hemostasis experts, it is very important that the report that is given to the clinician provides an interpretation of numerical results clarifying whether LA is present or absent, using the clear terminology “positive” or “negative” and avoiding expression such as “borderline” or “dubious”. Results of every diagnostic step must be reported, together with the employed local cut-offs. LA results should always be accompanied by those of anti-cardiolipin and aβ2-GP-I antibodies, to facilitate the assessment of risk profile. If laboratory diagnosis is positive, it should be indicated to repeat LA testing after 12 weeks [9] to avoid transient antibody positivity, such as that associated with infectious or phlogistic processes. A paradigmatic setting characterized by these findings is represented by children suffering from upper respiratory tract infection, diseases that are common in the youngest ages [108,109,110]. These patients can have a finding of LA as a consequence of an isolated prolonged aPTT observed in pre-surgery investigations for (adeno)tonsillectomy.

## 10. Discussion and Conclusions

Despite its name, LA actually increases thrombotic risk and is a significant risk factor for APS.

Clinicians, particularly those involved in hematology, rheumatology, internal medicine, and obstetrics, should have deep knowledge of LA diagnostic workup for several reasons:

- Diagnosis of APS: LA is one of the key criteria for diagnosing APS. Alongside other antibodies such as anticardiolipin antibodies and anti-β2 glycoprotein I antibodies, the presence of LA helps confirm the diagnosis of APS. Accurate diagnosis is crucial for managing patients with APS and preventing serious complications such as recurrent thrombosis and pregnancy complications.
- Risk assessment and management: Patients with LA are at an increased thrombotic risk, including deep vein thrombosis (DVT), pulmonary embolism (PE), and stroke. Identifying this risk allows clinicians to take appropriate preventive measures. This might include anticoagulant medications or lifestyle modifications to reduce the risk.
- Guiding treatment decisions: Knowing that a patient has LA can influence treatment decisions, especially for those who have already been diagnosed with autoimmune conditions such as SLE. The presence of lupus anticoagulant might impact the choice of medications and their dosages, as certain drugs can interact with the hypercoagulable state associated with APS.
- Pregnancy management: Pregnant individuals with LA are at a higher risk of miscarriages, stillbirths, and other pregnancy-related complications due to the increased clotting tendency. Clinicians need to closely monitor and manage these pregnancies to improve the chances of successful outcomes.
- Monitoring and follow-up: Patients with LA require regular monitoring and follow-up. Monitoring clotting factors and markers, as well as assessing the effectiveness of any prescribed anticoagulation therapy, helps ensure optimal patient care and prevents complications.
- Educational support: Clinicians play a crucial role in educating patients about their condition. Informing patients about LA and its implications empowers them to be proactive in their own healthcare, adhere to prescribed treatments, and make informed decisions.

Therefore, LA testing is indicated in all the clinical conditions listed in Table 2.

In summary, LA detection is an essential procedure to provide appropriate treatment, prevent complications, and offer informed guidance for managing both the underlying autoimmune disorder and the associated thrombotic risks of patients.

**Take home messages:**

- LA detection is based on functional coagulation assays that are characterized by poor standardization, difficulty in interpreting the results, and interference by several drugs commonly used in the clinical setting in which LA search is appropriate.
- LA detection is performed following a three-step approach that consists of a sequence of three coagulation procedures: screening, mixing, and confirmation.
- dRVVT is the most sensitive and specific test to detect LA.

## Figures and Tables

**Table 1:** Drugs that could be related to LA detection [10].

Drug Category	Specific Drug
Antiarrhythmics	Procainamide Quinidine/quinine
Antibiotics	Amoxicillin Penicillin Streptomycin Sulfasalazine
Anticonvulsants	Phenytoin Valproic acid
Antidepressants	Doxepin
Antihypertensives	Acebutolol Hydralazine Propranolol
Antipsychotics	Chlorpromazine Clozapine Fluphenazine Haloperidol Perphenazine
Immunosuppressants	Adalimumab Etanercept Infliximab
Immunotherapy	Interferon-α Interleukin-2

**Table 2:** Settings in which lupus anticoagulant (LA) search should be performed or could be considered, and the most appropriate timing of testing.

Situations in Which LA Testing Should Be Performed [9]	Situations in Which LA Testing Could Be Considered [9]	Timing of LA Testing [9,14]
Patients with unexplained prolonged aPTT with normal PT Patients younger than 50 y.o. with unprovoked venous thromboembolism Patients younger than 50 y.o. with ischemic stroke, transient ischemic attack, or other evidence of brain ischemia Patients younger than 50 y.o. with arterial thrombosis in other sites Unusual sites venous thrombosis Thrombosis of the microvasculature Recurrent venous thromboembolism while on adequate anticoagulation, not explained by cancer Pregnancy morbidity: fetal loss after 10 weeks, recurrent early (first trimester) miscarriages, prematurity (<34 weeks’ gestation) associated with severe (pre)eclampsia, HELLP syndrome, placental insufficiency (fetal growth restriction), stillbirth Systemic lupus erythematosus diagnostic workup	Patients younger than 50 y.o. with a venous thromboembolism associated with weak risk factors Patients younger than 50 y.o. with symptoms not included in Sydney criteria (e.g., cognitive disfunction, valvular heart disease in association with an autoimmune disease) Immune thrombocytopenia Livedo reticularis Isolated prolongation of aPTT as incidental finding	Results of LA testing during an acute thrombotic event or during acute phase should be interpreted with caution Results of LA testing during pregnancy should be interpreted with caution Ideally, LA testing should be performed in absence of anticoagulant therapy

HELLP syndrome: hemolysis, elevated liver enzymes, low platelet syndrome; PT: prothrombin time; aPTT: activated partial thromboplastin time.

**Table 3:** Lupus anticoagulant assays other than aPTT and dRVVT.

Test	Rationale	Pros/Cons	Uses/Recommendations
Dilute Prothrombin Time (dPT)	Activation of factor VII by thromboplastin; high dilution of thromboplastin in screening, low in confirmatory	Good sensitivity to LA; considerable variability in reagents	Alternative to aPTT for BSH, second-line test for CLSI
Kaolin Clotting Time (KCT)	Activation of intrinsic pathway by kaolin as contact activator; does not contain exogenous phospholipids	Lack of standardization; incompatibility with some analyzers using optical clot detection method	Largely used in the past, has been abandoned
Taipan Snake Venom Time (TSVT)	Activation of prothrombin by Taipan snake venom (Oscutarin C) in a phospholipid- and calcium-dependent way but independently from factor V	Good sensitivity to LA of the TSVT/ET combination, with less interference from anticoagulants (DOACs)	The TSVT/ET combination may be an option for LA testing in anticoagulated patients
Ecarin Time (ET)	Indian saw-scaled viper venom containing ecarin activates prothrombin independently from any cofactor, such as phosholipids		

(BSH: British Society of Hematology; CLSI: Clinical and Laboratory Standard Institute) [7,14,15,26,27,28,29,30,31,32,33,34,35,36].

**Table 4:** The effects of anticoagulant drugs on LA assays; the number of arrows indicate the width of the clotting time prolongation [64].

Anticoagulant	aPTT	SCT	dRVVT
UFH	↑/↑↑↑ (concentration dependent)	=(if neutralizers) ↑ (if exceeds or no neutralizers)	
LMWH	↑	=(if neutralizers) ↑ (if exceeds or no neutralizers)	
VKA	↑	↑	↑↑
Dabigatran	↑↑	↑	↑↑
Rivaroxaban	↑	↑↑	↑↑↑
Apixaban	=/↑ (assay dependent)	↑	↑
Edoxaban	=/↑ (assay dependent)	↑	↑

aPTT: activated partial thromboplastin time; SCT: silica clotting time; dRVVT: dilute Russell viper venom time; UFH: unfractionated heparin; LMWH: low-molecular-weight heparin; VKA: vitamin K antagonists.

**Table 5:** Main guidelines’ recommendations regarding LA testing in anticoagulated patients.

Guidelines	UFH	LMWH	VKA	DOACs
ISTH 2020 [9]	UFH and enoxaparin affect the dRVVT at supra-therapeutic anti-Xa levels Some reagents contain heparin neutralizers: it is important to verify the levels of heparins that are quenched in these reagents		Taipan/ecarin tests are less affected; recommendations for their general use awaits the provision of independent evidence Dilution of patient plasma into PNP is not a reliable solution (false negative or false positive may occur)	Taipan/ecarin tests are less affected by anti-FXa DOACs; recommendations for their general use awaits the provision of independent evidence If feasible to briefly interrupt DOAC anticoagulation, LA testing can be performed after checking the level of DOAC DOAC adsorption may be considered
		Samples should be taken, when feasible, at least 12 h after the last dose of LMWH was administered and as near as possible to the next dose		
ISTH SSC 2020 [14]	UFH clearly affects LA assays, especially aPTT-based, with false-positive screening and mixing results At low anti-FXa UFH activity levels, application of the three-step procedure does not produce false positives	Some brands of LMWH may result in sizeable prolongation of clotting tests and may affect LA detection	Although dilution of the test plasma into PNP is widely used, it is not robust enough to help make a diagnosis of LA, and both false-negative or false-positive results may occur	On a pragmatic empirical basis, LA testing may be undertaken at least 48 h after the last dose, and longer in patients with renal impairment, although DOAC levels should also be checked DOAC neutralizers can be considered
CLSI 2014 [15]	If possible, samples should not be screened with the aPTT or SCT unless treated with a heparin neutralizer Most dRVVTs contain a heparin neutralizer that permits testing; however, samples containing high UFH levels may give incorrect results	LMWHs may prolong the aPTT and therefore results should be interpreted with caution (however, in certain patients at high risk for APS and treated with LMWH, there is no alternative but to test in the presence of the drug)	If possible, VKA samples should not be screened with the aPTT because correct interpretation of test results is difficult Most patients also have prolonged SCT and dRVVT ∙	DOACs provide prolonged dRVVT results that show only partial correction in a screening mixing test
BSH 2012 [36]	LA tests should not be performed in patients receiving therapeutic doses of UFH because of potential erroneous results		LA testing is not recommended in patients receiving VKA Brief discontinuation of therapy for diagnostic purposes is not a high-risk strategy in most instances Performing testing on mixture of patient and normal plasma may be informative. Because of the dilution effect, negative testing in mixing studies does not exclude the presence of a LA Alternate assays to dRVVT can be considered Positive results from aCL or aB2GPI assays are sufficient for the diagnosis of APS	Not mentioned
	Low-dose subcutaneous UFH and LMWH have less effect on the dRVVT, and most commercial reagents contain heparin neutralizers sufficient to cover prophylactic doses. Positive results from aCL or aB2GPI assays are sufficient for the diagnosis of APS			

ISTH: International Society of Haemostasis and Thrombosis; ISTH SSC: Scientific and Standardization Committee (SSC) for lupus anticoagulant/antiphospholipid antibodies of the ISTH; CLSI: Clinical and Laboratory Standards Institute; BSH: British Society of Haematology; aCL: anticardiolipin antibodies; aB2GPI: anti-Beta2-Glicoprotein 1 antibodies; APS: antiphospholipid syndrome; aPTT: activated partial thromboplastin time; DOACs: direct oral anticoagulants; dRVVT: dilute Russell viper venom time; LMWH: low-molecular-weight heparin; SCT: silica clotting time; UFH: unfractionated heparin; VKA: vitamin K antagonists.

## Data Availability

Not applicable.
